# 

**Published:** 1864-06

**Authors:** 


### CHICAGO MEDICAL JOURNAL.

**Terms, $2.00 per Annum.**

### CONTENTS OF THE JUNE NUMBER.

PAGE.

ORIGINAL COMMUNICATIONS.

Some Account of the Prevailing Epidemic in the Northwest, variously designated, but usually popularly denominated "Spotted Fever." A Paper read before the Illinois State Medical Society, May, 1864. By J. Adams Allen, M. D., LL.D., Prof. Prin. and Prac. Med, and Clin. Med., Rush Med. College, &c.......... 241

Clinical Lectures on Diseases of the Eye. By E. L. Holmes, M. D., of Chicago, Lecturer on Diseases of the Eye and Ear in Rush Medical College, etc.—The Cornea—Chronic Inflammation.......... 258

Tænicide Properties of Pepo, with Report of a Case in which it was Successfully Used. By E. Ingals, M. D.......... 263

"Excito-Secretory."—Singular Misapprehension.......... 267

SELECTED.

Some Practical Observations on Small Pox and Vaccination, at Rock Island Prison Barracks, Rock Island, Ill. By R. M. Lackey, A. A. Surg. U. S. A., M. D.......... 267

Clinical Lectures on Diphtheria. By John T. Metcalfe, M. D., Prof. Practice of Medicine, University Med. College.......... 272

Editorial and Miscellaneous.......... 276

Book Notices and Reviews.......... 281

Obituary Notices.......... 284

